# Emotion Recognition as a Real Strength in Williams Syndrome: Evidence From a Dynamic Non-verbal Task

**DOI:** 10.3389/fpsyg.2018.00463

**Published:** 2018-04-05

**Authors:** Laure Ibernon, Claire Touchet, Régis Pochon

**Affiliations:** ^1^Centre de Recherche en Psychologie: Cognition, Psychisme et Organisations (EA 7273), Département de Psychologie, UFR Sciences Humaines et Sociales et Philosophie, Université de Picardie Jules Verne, Amiens, France; ^2^Laboratoire de Psychologie Cognition, Santé, Société (EA 6291), Université de Reims Champagne-Ardenne, Reims, France

**Keywords:** Williams syndrome, Down syndrome, emotion recognition, developmental trajectories, hypersociability

## Abstract

The hypersocial profile characterizing individuals with Williams syndrome (WS), and particularly their attraction to human faces and their desire to form relationships with other people, could favor the development of their emotion recognition capacities. This study seeks to better understand the development of emotion recognition capacities in WS. The ability to recognize six emotions was assessed in 15 participants with WS. Their performance was compared to that of 15 participants with Down syndrome (DS) and 15 typically developing (TD) children of the same non-verbal developmental age, as assessed with Raven’s Colored Progressive Matrices (RCPM; Raven et al., 1998). The analysis of the three groups’ results revealed that the participants with WS performed better than the participants with DS and also than the TD children. Individuals with WS performed at a similar level to TD participants in terms of recognizing different types of emotions. The study of development trajectories confirmed that the participants with WS presented the same development profile as the TD participants. These results seem to indicate that the recognition of emotional facial expressions constitutes a real strength in people with WS.

## Introduction

The diagnostic criteria of the American Association on Intellectual and Developmental Disabilities [AAIDD], 2013 refer to a social adaptation deficit affecting many people with intellectual disabilities (IDs). Consequently, the development of social skills constitutes a major avenue for interventions to better support individuals with ID. Moreover, Trentacosta and Fine (2010) carried out a meta-analysis that highlights the links between social competence and emotional knowledge: emotional knowledge has a positive impact on many aspects of social behavior. The study described here aims to better understand the development of emotion recognition capacities in people with Williams syndrome (WS), a neurodevelopmental disorder of genetic origin characterized by mild to moderate ID, a heterogeneous cognitive profile, and atypical social behavior, among other things. This social behavior is characterized by “hypersociability,” which manifests itself in a particular attraction to faces, high caring, and “niceness,” a lack of fear of strangers, and excessive talkativeness (Jones et al., 2000). According to Van Den Heuvel et al. (2016), individuals with WS are so eager to communicate that they will do anything to interact with other people, to the detriment of their sense of relationships. The parents of children with WS report that their children have this desire to interact with others, while also highlighting inappropriate social behaviors in interactions (Lough et al., 2016). For example, children and adolescents with WS preferentially seek to interact with adults or older people (Riby et al., 2014). At the linguistic level, this social behavior is reflected in a pragmatic deficit commonly referred to as “cocktail party speech” (Udwin and Yule, 1990), namely discourse that features correct articulation and appropriate intonation in association with impoverished content and the use of socially stereotyped expressions. Despite these communication problems, or characteristics, individuals with WS are especially attracted to their interlocutors’ faces (Riby and Hancock, 2008; Asada and Itakura, 2012), which, according to some authors, may explain their good facial recognition capacities (Mancini et al., 2006; Riby et al., 2008; Annaz et al., 2009; Dimitriou et al., 2014).

The hypersocial profile characterizing individuals with WS, and especially their attraction to human faces, could foster the development of their ability to recognize emotions, particularly positive emotions, which attract their attention most (Santos et al., 2010). Overall, people with WS have emotion recognition results similar to those of typically developing (TD) participants with the same developmental age (TD-DA), of participants with learning disabilities or ID, and participants with autism-spectrum disorder, whether the stimuli are static (Plesa-Skwerer et al., 2006; Lacroix et al., 2009) or dynamic (Gagliardi et al., 2003). In these various studies, the participants with WS presented response patterns similar to those of TD-DA participants. Although these results seem to rule out the hypothesis that these participants have a specific deficit affecting emotional facial expression recognition (EFER), it nevertheless appeared that there was some kind of developmental delay or atypical development. Indeed, except for the recognition of the emotion of happiness, their performance was inferior to that of TD participants with the same chronological age (TD-CA), TD participants matched for verbal age and, most surprisingly, participants with autism spectrum disorder, especially for the emotions of fear and sadness (Gagliardi et al., 2003; Plesa-Skwerer et al., 2006; Lacroix et al., 2009). According to Plesa-Skwerer et al. (2006), these results are the outcome of emotion processing at the superordinate, rather than subordinate, level: individuals with WS are better able to distinguish positive emotions than negative emotions, but they have difficulties differentiating between emotions with the same valence. In addition to this atypical emotion processing, the study by Gagliardi et al. (2003) revealed a correlation between performance on EFER tests and the intelligence quotient of participants with WS and indicated that this ability does not develop with age, which suggests that a permanent EFER delay is associated with this syndrome.

In the literature, WS is often compared to Down syndrome (DS) because both of these neurodevelopmental disorders of genetic origin are characterized by an equivalent moderate ID level. Their heterogeneous profiles are complementary, with weaknesses in the visuospatial domain for WS (e.g., Martens et al., 2008) and in the verbal domain for DS (e.g., Grieco et al., 2015). Like children with WS, children with DS have been described as more sensitive and attentive to other people’s emotions than other children with ID and as having a more marked interest in faces (Kasari and Freeman, 2001). Regarding their EFER capacities, several studies have shown deficits compared to TD-DA participants (Kasari and Freeman, 2001; Williams et al., 2005; Wishart et al., 2007; Pochon and Declercq, 2014), but certain recent studies using a non-verbal protocol and/or dynamic stimuli did not show any difference at equivalent developmental ages (Pochon and Declercq, 2013; Channell et al., 2014).

To the best of our knowledge, only two studies have compared the EFER performance of people with WS and people with DS. Porter et al. (2007) administered the Diagnostic Analysis of Non-verbal Accuracy Scale – second edition (DANVA-2; Nowicki and Duke, 1994) to four groups of 20 participants each: a WS group (*m* = 16.2 years), a DS group (*m* = 16.5 years), a TD-DA group (*m* = 4.11 years), and a TD-CA group (*m* = 15.11 years). The DANVA-2 comprises four subtests designed to assess recognition of the facial and vocal expressions of emotions (happiness, sadness, anger, and fear) by children and adults. The WS group scored higher on this test than the DS group and similarly to the TD-DA group but lower than the TD-CA group. In both the WS and DS groups, happiness was generally better recognized than negative emotions. The DS group made more errors in the recognition of negative emotions than the WS group. More recently, Martínez-Castilla et al. (2015) carried out an analogous study (WS: *m* = 12.3 years; DS: *m* = 12.6 years; TD-CA: *m* = 12.8 years; TD-DA: *m* = 4.11 years) using a verbal task: the Animated Full Facial Expression Comprehension Test (AFFECT; Gagliardi et al., 2003). Participants had to label basic emotional expressions (happiness, sadness, anger, fear, and disgust), which were presented by means of animated faces. The results of that study confirmed those of Porter et al. (2007): the participants with WS performed better than the group with DS, similarly to the TD-DA group, and worse than the TD-CA group. However, these results concern all the emotions and not just happiness. It therefore appears that happiness is not the only emotion the recognition of which is preserved in participants with WS. Nevertheless, the analysis of cross-sectional developmental trajectories revealed an atypical development profile in both groups with ID. As Gagliardi et al. (2003) had already suggested, in participants with both WS and DS, EFER capacities speedily reach a ceiling level and then stop developing with increased age.

In those two intersyndrome studies, the authors opted for a verbal protocol in which participants had to understand an emotional lexicon. However, the possible lag with regard to verbal aptitudes was not taken into consideration since participants with WS and DS were matched on the basis of their performance on a global cognitive evaluation. Nevertheless, linguistic abilities constitute an essential component in emotion recognition (Salmon et al., 2013) and language skills could facilitate EFER in individuals with WS (Lacroix et al., 2009). To our knowledge, no study of emotion recognition in participants with WS has used a non-verbal protocol, which may have contributed to overestimating their competence. In a verbal EFER task, participants with WS could be at an advantage because of their good language skills, whereas participants with DS, who may be disadvantaged by their language problems (e.g., Grieco et al., 2015), perform poorly. This hypothesis is supported by the results of studies that showed no difference between the scores of participants with DS and those of TD-DA participants on a non-verbal EFER task (Pochon and Declercq, 2013; Pochon et al., 2017).

This study was designed to contribute to a better understanding of EFER capacities in people with WS, by comparing their performance with that of people with DS and of TD-DA. Our first objective was to compare the EFER capacities of a group of participants with WS with that of a group of participants with DS and a group of TD-DA participants. We created an original test, based on video clips, which does not use emotional vocabulary and does not require participants to respond verbally. This test was chosen because people with DS have language deficits (e.g., Grieco et al., 2015); thus, giving them a verbal task could cause difficulties for them and contribute to underestimating their EFER competences. Conversely, several studies have concluded that language has a positive impact on emotion recognition (Salmon et al., 2013), and thus the use of emotional vocabulary could create an advantage for participants with WS and thereby contribute to overestimating their emotion recognition abilities. The value of this task, which we will call a “non-verbal” one, in addition to the use of more ecological stimuli such as video clips, as recommended by Moore (2001), is that it does not solicit emotion recognition by means of an emotional lexicon. Given the results of previous studies with verbal EFER tests, we formulated the following hypothesis: EFER really is a strength in WS and participants with WS will perform better than participants with DS and similarly to TD-DA participants, in accordance with previous studies that used verbal tasks (Gagliardi et al., 2003; Porter et al., 2007; Lacroix et al., 2009; Martínez-Castilla et al., 2015). Alternatively, if the EFER competences of participants with WS reported in previous studies were essentially based on their preserved language skills, their performance on a non-verbal task should be comparable to that of participants with DS and poorer than that of TD-DA participants.

The second objective of this study is to refine EFER competence profiles based on the type of emotion (happiness, sadness, fear, anger, disgust, surprise). In most studies of EFER, emotions are classified in two superordinate categories: happiness vs. non-happiness (i.e., sadness, fear, anger, disgust, and surprise). Several studies have shown that individuals with WS are better at recognizing happiness than non-happiness emotions (Plesa-Skwerer et al., 2006; Porter et al., 2007; Lacroix et al., 2009). This outcome could be partly due to the fact that most studies have used only one positive but several negative emotions. In accordance with past studies, we expected that the results for the recognition of happiness of participants with WS would be better than those of the group with DS and the TD-DA group. Concerning non-happiness emotions, few data were available on well-recognized emotions, and those data came from studies that used verbal protocols. Thus, we could not make any predictions regarding the recognition of those emotions.

## Materials and Methods

### Participants

Ethical approval was not required for this study, according to the national and institutional guidelines. This study was carried out in accordance with the recommendations of French law that written informed consent be obtained from all subjects. All participants gave their written agreement to participate, and their parents and/or legal guardians were informed of the objectives of the study, the nature of the tasks that would be administered, and the fact that they could withdraw their agreement at any time. Their informed consent was received in writing in accordance with the Declaration of Helsinki. The medical or social and academic authorities were also informed and agreed that the students could take part, since most of the meetings took place at the educational institution.

A total of 45 French-speaking children, adolescents, and young adults divided into three groups (two ID groups and one control group) took part in this study. The first group (henceforth, WS group) was made up of 15 participants with WS; their mean age was 15.1 years (6.2–27.2 years old). The fluorescence *in situ* hybridization (FISH) technique revealed that all participants in the WS group were positive for 7q11.23 microdeletion. The second group (henceforth, DS group) was composed of 15 participants with DS who had a mean age of 16.1 years (10.7–23.9 years old). The diagnosis of trisomy 21 was confirmed by the medical teams at the institutions where these young people were being followed up. There were no statistically significant differences between the two ID groups regarding the age factor [*t*(28) = 0.54, *p* = 0.60]. The third group (henceforth, TD-DA group) comprised 15 TD participants with a mean age of 4.5 years (3.6–5.1 years old). These TD children were selected from a sample of 69 children aged 3.5–10 years who had completed the experimental tasks in a previous study (Pochon et al., 2015).

The DS group and the TD-DA group were formed by individual matching with the participants in the WS group for non-verbal reasoning, as assessed with Raven’s Colored Progressive Matrices (RCPM; Raven et al., 1998). We avoided matching participants by means of a task that is especially relevant for emotion recognition because using such a measure to match groups would run the risk of over-controlling. Since the tests were non-verbal, we chose the RCPM as a preliminary measurement of the three groups’ development level. There was no significant difference between the three groups in this regard [*F*(2,42) = 0.08, *ns*]. Given that the ability to efficiently process faces can impact success on EFER tasks, the participants were also given the long version of the Benton Facial Recognition Test (Benton et al., 1983). This is a standardized test that assesses the ability to identify unfamiliar faces. A significant group effect appeared [*F*(2,42) = 6.32, *p* = 0.004, $\eta_{p}^{2}$ = 0.23]. *Post hoc* analyses (Tukey’s test) revealed that the WS group scored better than the DS group (*p* = 0.005) and the TD-DA group (*p* = 0.026), while the DS and TD-DA groups had similar results.

**Table 1** summarizes the characteristics of each group.

**Table 1:** Descriptive characteristics of the WS, DS, and TD-DA groups.

	Group					
	Williams syndrome		Down syndrome		Typically developing	
Variables	Mean	*SD*	Mean	*SD*	Mean	*SD*
Gender (M/F)	8/7	–	9/6	–	6/9	–
Chronological age – mean	180.80	74.20	192.67	42.87	52.87	6.71
Chronological age – range	74–326	–	127–285	–	42–61	–
RCPM raw score	17.20	4.84	16.8	5.87	17.53	4.47
BFRT score	36.80	5.58	31.53	3.42	32.53	3.58

*N = 15 in each group. Ages are reported in months. RCPM, Raven’s Colored Progressive Matrices; BFRT, Benton Facial Recognition Test.*

### Design and Procedure

Two experimental tasks with similar constructions were administered to participants. These tasks had been examined in a study of children aged 3–11 years old to ensure that they were sensitive to advancing age and to collect typical developmental data (Pochon et al., 2015). The material was the same as was used and described in detail in Pochon et al.’s (2017) study.

#### Control Task

Six familiar objects were used for this task: a small plastic bottle, a ceramic bowl, a metal cooking pot, a stemmed glass, a plastic citrus juicer, and a plastic kitchen spatula. Each one was used three times as a target. Each object was presented in short video clips (3.2 s) in which it was struck by another object: nine times with a large wooden spoon and nine times with a large metal spoon, which produced different sounds. The objects were hit in three different ways: three knocks, two double knocks or three double knocks. Each method was used six times. During each presentation, two video clips were presented simultaneously, depicting the same hitting object and the same hitting method; the only difference was the object that was hit (one target object and one distractor). Participants were asked to match the sound they heard with the corresponding video clip. They responded by manually pointing at the screen of a portable computer (15″, resolution of 1366 pixels × 768 pixels). The video clips were shown side by side with a small space between them and were played simultaneously after a target was presented to encourage the participant to gaze at the center of the screen. They were played in a loop until the participant responded. The maximum score on this task was 18 (six target objects presented three times). The use of a control task involving the same cognitive demands as the emotional task ensured that any difficulties encountered in expression recognition were due not to the characteristics of the task itself but to an impairment in emotional information processing (Moore, 2001).

#### Emotional Task

Six basic emotional facial expressions were presented during this task: happiness, sadness, anger, disgust, surprise, and fear. Each one was the target emotion three times. Each time, the emotions were expressed by the same professional actor (nine times by a man, nine times by a woman) with only the head and shoulders visible. Both actors were trained to express these emotions as needed by the task, and the video clips used were selected from numerous takes: they had to be correctly identified in 95% of cases by 20 non-expert adults aged 20–40 years. The actors alternately spoke three sentences in French that either had non-emotional content (e.g., “The bottle is on the table”) or were made up of non-words (e.g., “Cognogo tiketou”). Each sentence was used six times and was spoken with the appropriate prosody and facial expression for the relevant emotion. For each presentation, the same actor speaking the same sentence appeared in both video clips; only the emotion expressed by his/her face and voice differed. To respond correctly, participants had to match the prosody of the sentence they heard with the corresponding facial expression. The stimuli were presented with a method identical to that used in the control task. The maximum score on this task was also 18 (six target emotions presented three times).

There was a total of 36 presentations: 18 presentations of familiar objects in motion and 18 presentations of actors’ faces talking. A single soundtrack, corresponding to the target video clip, was played with a time lag (desynchronization) so participants could not use the synchronization of the sound with the object’s movements or the actor’s lips as a cue.

Participants were tested in a quiet, familiar room at their health care institution or school or at home. The tasks took a total of 70–90 min and were divided among three sessions lasting 20–30 min each so the results would not be affected by fatigue, boredom, or concentration problems. The administration of the experimental task was divided into four blocks: one learning block and three experimental blocks. Each block comprised 12 items, six from the control task and six from the emotional task, presented alternately. A pause after each block allowed experimenters to chat with participants and keep them motivated. The purpose of the learning block was to teach participants the task and ensure that they understood it. If the participant still could not understand the task at the end of this block, the task administration was interrupted. The initial instructions for the control items were as follows: “Listen carefully to me. Now, when I press this button, you’ll see two short films, one on the left and the other on the right [the examiner shows the locations on the blank screen]. At the same time as you’re watching these two little films, you’ll hear a sound. You have to point your finger at the film that goes with the sound we hear – the one on the left or the one on the right. Do you understand? Now, we’re starting – are you ready? Watch this target closely.” For the emotional items, the initial instructions were as follows: “Listen carefully to me. Now, when I press this button, you’ll see two short films, one on the left and the other on the right [the examiner shows the locations on the blank screen]. At the same time as you’re watching these two little films, you’ll hear someone talking. You have to point your finger at the film where the person is talking – the one on the left or the one on the right. Do you understand? Now, we’re starting – are you ready? Watch this target closely.” Throughout the task, no emotion words were used. It very quickly became unnecessary to repeat the instructions in full.

## Results

After comparing the global results at the control and emotional tasks, subsequent analyses involved two approaches: first, the individual matching method in which an analysis of variance (ANOVA) was used to compare the emotion recognition abilities of participants with WS, participants with DS, and TD-DA participants. The second approach was the study of cross-sectional developmental trajectories related to emotion recognition, first for all emotions and then for each one separately.

### Comparison of the Results of Groups Matched for Non-verbal Reasoning (RCPM)

The normality of distributions for each variable studied was tested for each group using one-sample Kolmogorov–Smirnov tests. The distribution was normal for the overall results on the control and emotional tasks and for each emotion in the emotional task, which made it possible to carry out ANOVAs followed by *post hoc* tests (Tukey’s test).

#### Global Results on Control and Emotional Tasks

**FIGURE 1:**
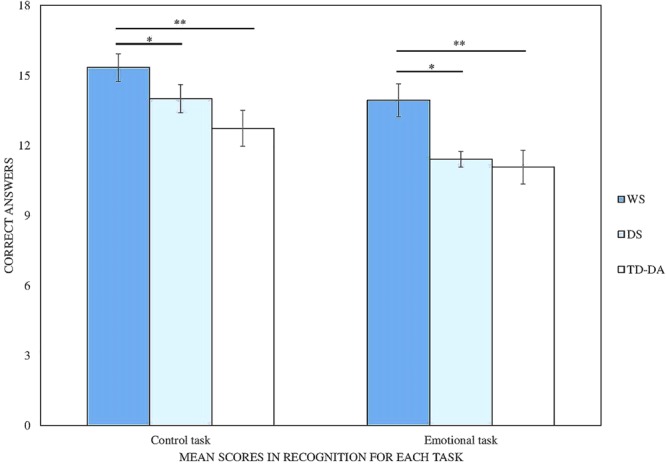
Mean scores for each task in RCPM-matched groups (maximum score = 18). WS, Williams syndrome; DS, Down syndrome; TD, typically developing. ^∗^*p* < 0.05, ^∗∗^*p* < 0.01.

The results on the two experimental tasks (**Figure 1**) were analyzed with a mixed-design ANOVA with Group (WS, DS, TD-DA) as between-subjects variable and Task (control, emotional) as within-subjects variable.

The analysis of the results revealed a significant Group effect [*F*(2,42) = 7.20, *p* = 0.002, η^2^ = 0.26]. *Post hoc* comparisons showed that the WS group obtained better results than the DS group (*p* = 0.03) or the TD-DA group (*p* = 0.002), whereas the DS and TD-DA groups performed at equivalent levels. Participants generally performed better on the control task than the emotional task [*F*(1,42) = 20.53, *p* < 0.001, η^2^ = 0.33]. Nevertheless, this difference was only significant for the DS (*p* < 0.001) and TD-DA groups (*p* < 0.05), and not for the participants with WS.

#### Analysis of Response Profiles on the Emotional Task

To identify response profiles on the emotional task, we carried out a 3 (Group) × 6 (Emotion) repeated-measures ANOVA (**Table 2**). We found a main effect of Group [*F*(2,42) = 6.58, *p* = 0.003, η^2^ = 0.238], and of Emotion [*F*(3.9,163.81) = 10.61, *p* = 0.000, η^2^ = 0.202], and a Group × Emotion interaction [*F*(7.8,163.81) = 3.54, *p* = 0.001, η^2^ = 0.144]. The study of scores for each emotion does not reveal any significant differences between the three groups regarding the recognition of happiness and surprise. On the other hand, the participants with WS were better at recognizing fear (*p* < 0.05) and disgust (*p* < 0.001) than participants with DS and performed better than the TD-DA group at recognizing anger (*p* < 0.05). As for the DS group, they scored better than the TD-DA participants for the recognition of sadness (*p* < 0.01) but worse for disgust (*p* < 0.05). Intragroup comparisons revealed no significant differences between recognition of the six basic emotions in participants with WS and TD-DA participants. However, participants with DS recognized happiness better than fear, anger or disgust (*p* < 0.05 at least); in fact, disgust was the least recognized emotion.

**Table 2:** Scores for recognition of individual emotions in RCPM-matched groups.

		Group		
		Williams syndrome	Down syndrome	Typically developing
Emotions	Maximum score	Mean (*SD*)	Mean (*SD*)	Mean (*SD*)
Happiness	3	2.6 (0.63)	2.7 (0.49)	2.27 (0.6)
Fear	3	2.33 (0.61)	1.53 (1.06)	2.2 (0.86)
Anger	3	2.4 (0.63)	1.93 (0.59)	1.6 (0.83)
Disgust	3	2.13 (0.99)	0.8 (0.86)	1.53 (0.92)
Sadness	3	1.87 (1.19)	2.2 (0.68)	1.27 (1.1)
Surprise	3	2.6 (0.51)	2.27 (0.8)	2.2 (0.68)
Total	18	13.93 (2.71)	11.43 (1.3)	11.07 (2.79)

*Standard deviations are shown in parentheses.*

### Study of Developmental Trajectories for Emotion Recognition

**FIGURE 2:**
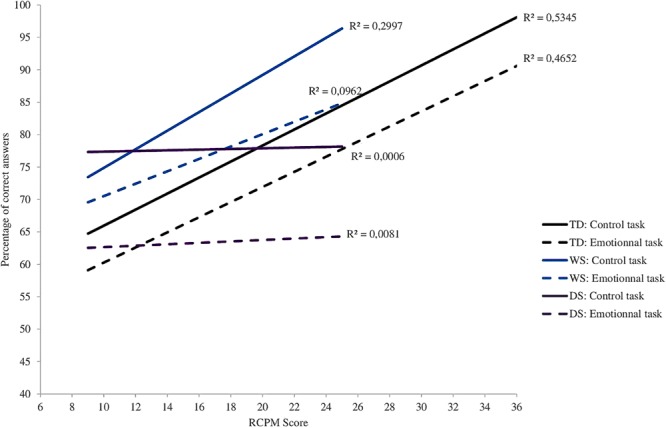
Cross-sectional developmental trajectories for the experimental tasks as a function of RCPM score. WS, Williams syndrome; DS, Down syndrome; TD, typically developing.

For the analyses of the developmental trajectories, the entire sample of TD-DA children (*N* = 69) was used, and consequently typical developmental trajectories were established on the basis of chronological ages ranging from 3.5 to 10 years. This made it possible to cover an essential segment of the ages in which recognition of the basic emotions develops (Herba and Phillips, 2004). First, the developmental trajectories for emotion recognition based on RCPM score were determined^1^. **Figure 2** shows the development of performance for the three groups on the two experimental tasks (control and emotional). Within each group, the slopes representing each task are almost parallel. The aim is to characterize the WS group’s trajectory with reference to typical development. As Thomas et al. (2009) suggested, comparisons were made using an analysis of covariance (ANCOVA), with score on the experimental task as the dependent variable, group as the categorical variable, and RCPM score as the covariate.

#### Trajectories for the Emotional Task

In the case of the participants with WS and TD-DA participants, the slopes representing scores on the emotional task are relatively similar and they increase regularly with increases in RCPM score. In the participants with DS, on the other hand, the essentially flat slope seems to indicate that performance on this test does not improve with RCPM score.

The ANCOVA indicates that there is no Group effect at the start of the trajectory [*F*(2,93) = 1.26, *p* = 0.29, $\eta_{p}^{2}$ = 0.026] and that overall the relationship between success on the emotional task and RCPM score is significant [*F*(1,93) = 7.41, *p* = 0.007, $\eta_{p}^{2}$ = 0.073]. Nevertheless, this relationship does not differ from one group to another since the interaction between group and RCPM score does not achieve significance [*F*(2,93) = 2.01, *p* = 0.14, $\eta_{p}^{2}$ = 0.041].

**FIGURE 3:**
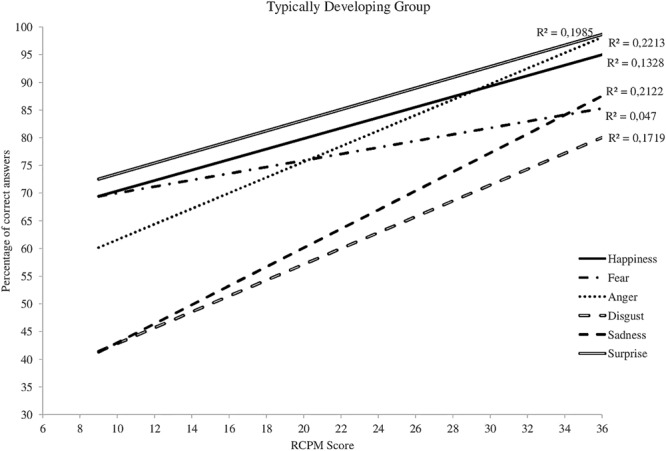
Cross-sectional developmental trajectories for the six basic emotions as a function of RCPM score, TD-DA group.

**FIGURE 4:**
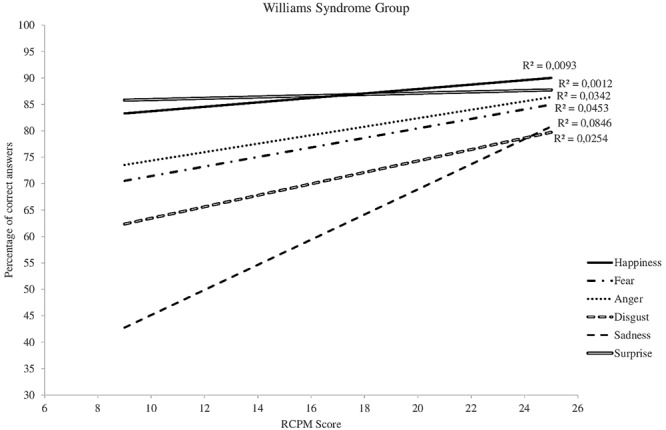
Cross-sectional developmental trajectories for the six basic emotions as a function of RCPM score, WS group.

**FIGURE 5:**
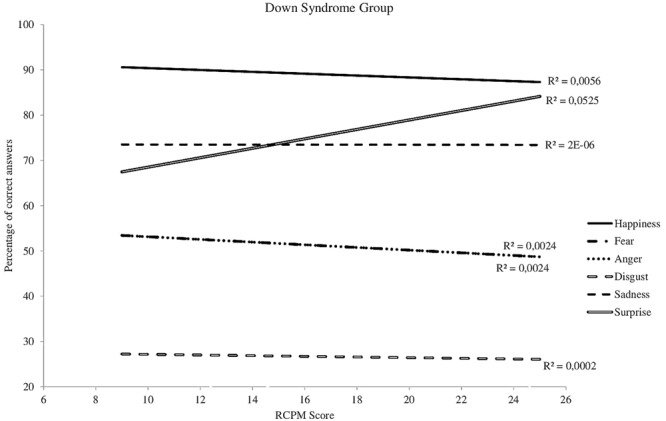
Cross-sectional developmental trajectories for the six basic emotions as a function of RCPM score, DS group.

Differences between the three groups also appear when one studies the recognition of each emotion separately. The slopes characterizing the increase in recognition abilities as a function of RCPM score are relatively similar for the WS and TD-DA groups (**Figures 3, 4**), whereas there is no change for the participants with DS (**Figure 5**). For the WS group, scores based on type of emotion increase progressively except for happiness and surprise, which were already well-recognized at the onset. The linear regression analysis reveals that, for the TD-DA group, emotion recognition improves significantly with the increase in RCPM score (*ps* < 0.002), except in the case of fear, for which they did not achieve the significance level (*p* = 0.072). For the WS and DS groups, no gradient differs significantly from 0, indicating that RCPM score cannot be considered as a reliable predictor of recognition of the six basic emotions in the groups with disorders.

#### Trajectories for the Control Task

The ANCOVA shows a significant effect of Group [*F*(2,93) = 3.03, *p* = 0.05, $\eta_{p}^{2}$ = 0.061] but no significant interaction between score on the control task and score on the RCPM [*F*(2,93) = 2.82, *p* = 0.065, $\eta_{p}^{2}$ = 0.057].

At the onset, the WS and DS groups had a significantly higher percentage of successes than the TD-DA group. In the participants with WS and the TD-DA participants, clear progress on the control task can be seen in association with the increase in RCPM score (WS: *R*^2^ = 0.3, TD-DA: *R*^2^ = 0.53); for the highest scores, performance tended to reach the ceiling level. On the other hand, this kind of change is not seen in the DS group, for which the slope remains horizontal. This is confirmed by the regression analysis, which shows no correlation between success on the control task and RCPM score in the DS group (*R*^2^ = 0.00).

## Discussion

In our test, the performance of participants with WS was significantly higher that of participants with DS, and also higher than that of the TD-DA participants. These results are consistent with the findings of previous cross-syndrome studies (Porter et al., 2007; Martínez-Castilla et al., 2015). However, they do not corroborate the results of the studies by Gagliardi et al. (2003), Plesa-Skwerer et al. (2006), and Lacroix et al. (2009), in which participants with WS performed comparably to TD-DA participants. These differences were not observed for all emotions: no difference emerged between the three groups for the emotions of happiness and surprise. Thus, these findings only partially corroborate those of Martínez-Castilla et al.’s (2015) study, which used dynamic stimuli and emotional productive vocabulary and in which the results of participants with WS were identical to those of the TD-DA group and better than those of participants with DS for all the emotions tested. These differences are probably attributable to the use of a dynamic non-verbal protocol. For intragroup profiles, the participants with WS presented the same success pattern as the TD-DA group for all the emotions studied; in both these groups, no difference between emotions appeared, contrary to the DS group. Thus, in contrast to previous studies (Plesa-Skwerer et al., 2006; Porter et al., 2007; Lacroix et al., 2009), participants with WS did not recognize happiness better than other emotions.

More importantly still, in this non-verbal task, the participants with WS obtained similar (compared with participants with DS) or even higher (compared with TD-DA participants) results than in previous studies that used a verbal protocol (Gagliardi et al., 2003; Porter et al., 2007; Lacroix et al., 2009; Martínez-Castilla et al., 2015). In addition, while the participants with DS and the TD-DA participants performed the control task better than the emotional task, this was not true of the participants with WS, who were equally successful at both tasks. It therefore appears that the recognition of emotional facial expressions is a true strength in people with WS and not merely a consequence of their language skills. Several studies have concluded that language has a beneficial impact on emotion recognition (Salmon et al., 2013), so the use of emotional vocabulary could have given participants with WS an advantage in past studies and contributed to overestimating their emotion recognition competences. That cannot be the case in this study, in which EFER was studied with a non-verbal task. Thus, any use of emotional vocabulary was ruled out. Nevertheless, this result must be considered with caution. Since we did not have access to participants’ problem-solving strategies, we cannot be certain that they were not making use of the emotional label when they gave their responses. If that were the case, participants with WS would be engaging in a kind of double coding (non-verbal and verbal).

### Discussion of Results of the Study of Cross-Sectional Developmental Trajectories

The study of cross-sectional developmental trajectories confirmed and refined the findings of the study of matched groups. Indeed, the participants with WS presented the same developmental profile as the TD-DA participants. This finding contradicts that of Martínez-Castilla et al. (2015), who used age rather than cognitive level as covariate. Nor does it corroborate the results of Gagliardi et al.’s (2003) study, which showed that EFER capacity did not develop with age. In participants with DS, however, the maximum developmental level is achieved early, and once it is achieved, performance remains static. It is as though the development of emotion recognition capacities had stopped or was happening outside the scope of influence of non-verbal reasoning capacities.

The second important result is that, in the groups with disorders, success on the RCPM is not a valid predictor of non-verbal emotion recognition, whereas it is a very satisfactory predictor for TD children. We chose non-verbal RCPM level as a preliminary measure because the experimental tasks were non-verbal and because the psychometric and developmental qualities of the RCPM mean that the tool is widely used with participants with developmental disorders (Facon et al., 2011). Although the lack of any link between results on the RCPM and on the experimental tasks is very clear in the participants with DS, it is much less clear in those with WS. In the latter group, we saw that performance on the two tasks and on the recognition of emotions in the emotional task improved with an increase in RCPM score, even though the increase was not significant.

### General Discussion

Our study has highlighted several interesting results. First of all, the emotion recognition abilities of participants with WS, as tested non-verbally, are better than those of participants with DS and, more importantly, better than those of TD-DA children. In the last 10 years, many reviews (e.g., Brock, 2007; Mervis and Becerra, 2007) and meta-analyses (Martens et al., 2008) have suggested that the capacities of participants with WS were indeed surprising but actually corresponded only to what one might expect given their generalized cognitive deficit. However, the results obtained by the participants with WS in our study seem to show that EFER is a real strength for this population. Secondly, the study of cross-sectional developmental trajectories revealed similar development profiles for participants with WS and TD participants. In fact, the performance of the participants with WS was so good that it seems impossible not to acknowledge that they have the same EFER competences as TD children with the same developmental age.

Nevertheless, our study does have certain limitations. The lack of correlation in the two groups with disorders between their RCPM level and their success on the experimental tasks remains surprising and should be investigated in more depth. We opted for this matching method in view of the non-verbal nature of our test, in accordance with Moore’s (2001) recommendations. It is possible that this was not sufficient. Moreover, it is also possible that emotion recognition might be closely linked to language abilities. Without having access to the way in which participants approached the task, we cannot claim that the participants with WS did not call upon their linguistic competences. A broader emotional lexicon, by instance, is a significant advantage for the categorization of facial expressions (Russell and Widen, 2002; Widen and Russell, 2004). The language of participants with WS is better than that of participants with DS, and language plays a critical role in the labeling of emotions (Salmon et al., 2013). Consequently, participants with WS may quite simply have more experience with labeling emotions than participants with DS with the same CA and/or DA. It would therefore be valuable to replicate this work using another matching method, such as level of receptive vocabulary. This type of study would also be important for clinicians. If language plays such a fundamental upstream role in the development of emotional competences, it is important to offer therapeutic and educational interventions as early as possible to avoid the entrenchment of the kinds of inappropriate social behaviors often described in individuals with developmental delays (e.g., Dykens, 2000; Crnic et al., 2004; Cook and Oliver, 2011).

Our research has confirmed that cross-syndrome studies are crucial in gaining a better understanding of developmental disorders. The comparison of the performance of the participants with WS and those with DS enables one to more effectively determine whether an atypical approach to emotion recognition is due to the intrinsic characteristics of WS (specific deficit hypothesis) or is the consequence of a global delay in cognitive development (non-specific deficit hypothesis). In this study, the explanation may rely on the characteristics of the two syndromes: a sociable profile in participants with DS vs. a hypersocial one in individuals with WS. People with WS exhibit a strong attraction to human faces, excessive volubility and a desire to interact with other people that could favor the development of their emotion recognition abilities.

## Author Contributions

LI: analysis and interpretation of the data and writing the manuscript. CT: acquisition of the data and drafting the manuscript. RP: conception and design of the task, analysis and interpretation of the data, and writing the manuscript.

## Conflict of Interest Statement

The authors declare that the research was conducted in the absence of any commercial or financial relationships that could be construed as a potential conflict of interest.

## Footnotes

1 We examined cross-sectional developmental trajectories for emotion recognition based on chronological age. The pattern of significant and non-significant results was the same as when RCPM scores were entered as predictors of accuracy. In the ID groups, chronological age is not a valid predictor of non-verbal emotion recognition, whereas it is a very satisfactory predictor for TD children.
